# 2D and 3D Classification Systems for Adolescent Idiopathic Scoliosis: Clinical Implications and Technological Advances

**DOI:** 10.1111/os.14362

**Published:** 2025-01-18

**Authors:** Wenqing Wei, Liang Cheng, Yating Dong, Tianyuan Zhang, Yaolong Deng, Jiale Gong, Fang Xie, Junlin Yang

**Affiliations:** ^1^ Spine Surgery Center, Xinhua Hospital Shanghai Jiao Tong University School of Medicine Shanghai China; ^2^ School of Health Science and Engineering University of Shanghai for Science and Technology Shanghai China; ^3^ Shanghai Marine Diesel Engine Research Institute Shanghai China

**Keywords:** 3D classification, adolescent idiopathic scoliosis, King classification, Lenke classification, PUMC classification, Rigo classification, Schroth classification

## Abstract

Classification systems for Adolescent Idiopathic Scoliosis (AIS) play an important role in guiding both surgical planning and conservative treatments. Traditional 2D classification systems, such as the Lenke, King and Lehnert‐Schroth classifications, have been widely used for the clinical diagnosis and treatment of scoliosis. However, with the growing understanding of the three‐dimensional nature of scoliosis and advancements in 3D reconstruction technologies, 3D classification systems are gaining increasing attention. This paper reviews the current applications, advantages, and limitations of different 2D and 3D classification systems, focusing on their clinical significance in treatment planning. While 3D classification systems offer clear advantages in capturing the complexity of spinal deformities, their clinical implementation faces challenges such as high costs and technical complexity. Additionally, studies show that computer‐assisted technologies, artificial intelligence can significantly improve the accuracy and consistency of classification systems, reducing human errors. The paper also explores the future directions of classification system development, emphasizing the potential of combining 2D and 3D technologies and the impact of these advancements on personalized scoliosis treatment.

## Graphical abstract

This study compares traditional 2D scoliosis classification systems (e.g., King, Lenke, and PUMC) with emerging 3D systems. It highlights the limitations of 2D methods and the advantages of integrating 3D models for more precise treatment planning and outcomes. The analysis shows that while 3D classification offers high reliability and comprehensive insights, challenges like complex modeling and clinical integration remain.

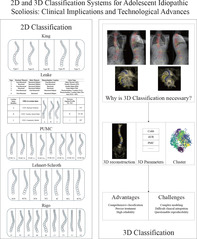

## 1 Introduction

Idiopathic scoliosis (IS) is a musculoskeletal disorder characterized by a three‐dimensional deformity of the spine, occurring at different ages: Infantile Idiopathic Scoliosis (IIS) manifests from birth to age 3, Juvenile Idiopathic Scoliosis (JIS) from ages 4 to 10, and Adolescent Idiopathic Scoliosis (AIS) from age 10 to the end of adolescence [1, 2]. The etiology of AIS remains unclear, with genetics, biomechanics, and neurology links [3]. The prevalence of AIS ranges from 0.47% to 5.2%, with a significantly higher incidence in females compared to males [4]. Additionally, the incidence of right thoracic curve is higher than that of left thoracic curve [5]. Untreated scoliosis has a risk of further curve progression, potentially leading to back pain, disability, or lung impairment, depending on the severity of the curve.

Since the Scoliosis Research Society (SRS) introduced treatment recommendations based on the severity of AIS, the treatment options now encompass a range of approaches, including observation, bracing, and surgical intervention [6]. For patients with mild scoliosis, with a Cobb angle less than 20°, it is recommended to undergo an X‐ray or clinical examination every 6 months until skeletal maturity. Early braces focused on preventing the progression of the curve. However, with a deeper understanding of the biomechanics of bracing, current designs aim to redistribute mechanical stress through the application of external forces [7]. This promotes vertebral remodeling, reduces spinal curvature, and aims to restore anatomical alignment of the spine. For patients with moderate scoliosis, between 20° and 40°, especially those who are skeletally immature with a Risser grade of 0–1, bracing is recommended. Some patients with Cobb angle greater than 45° have achieved good outcomes with bracing treatment [8]. The primary purpose of surgery is to eliminate the risk of further curve progression, thereby minimizing the long‐term risk of cardiopulmonary complications [9]. For severe or rapidly progressing scoliosis with a Cobb angle exceeding 40°–50°, surgical treatment becomes a relatively preferred option. The goal is to correct spinal balance and prevent further progression through stable fusion and internal fixation. Pedicle screw fixation is considered the most effective method for spinal deformity correction. In certain cases, end hooks may also be used to enhance the fusion outcome [10].

The AIS classification systems have also undergone development along with advancements in treatment approaches. The earliest classification of IS was proposed by Schulthess in 1905, categorizing scoliosis based on the location of the apex into thoracic, lumbar, thoracolumbar, cervicothoracic, and combined types, this system was later revised and refined by Ponseti and Friedman in 1950 [11, 12]. Together with the concepts of major and minor curves, as well as structural curves described by John, these contributions laid the foundation for modern scoliosis classification [13].

Up to now, representative classifications include:

In 1983, King et al. proposed a classification based on Harrington instrumentation surgery, known as the King classification [14]. Lenke developed a new classification system for IS in 2001. This classification remains the most widely used system to date [15]. In non‐surgical scoliosis classification, the well‐known systems include the Schroth classification and the Rigo classification [16, 17]. Rigo classification emphasizes the design of braces, while Schroth classification focuses on Schroth exercises. Given that scoliosis is a three‐dimensional deformity of the spine, SRS introduced the concept of 3D in 1994 and began studying the clinical relevance and impact of 3D analysis of scoliosis deformities [18].

In both surgical and non‐surgical fields, classification methods and their corresponding treatment strategies play a crucial role. Detailed classification methods and corresponding treatment strategies play a vital role in personalized and optimal treatment plans. Studies focus on innovating classification systems and treatment methods for AIS, aiming to significantly improving treatment outcomes.

This study aims to provide a systematic review, comprehensively analyzing AIS classification and related research to offer insights and guidance for future research and clinical advancements.

## 2 Materials and Methods

**FIGURE 1:**
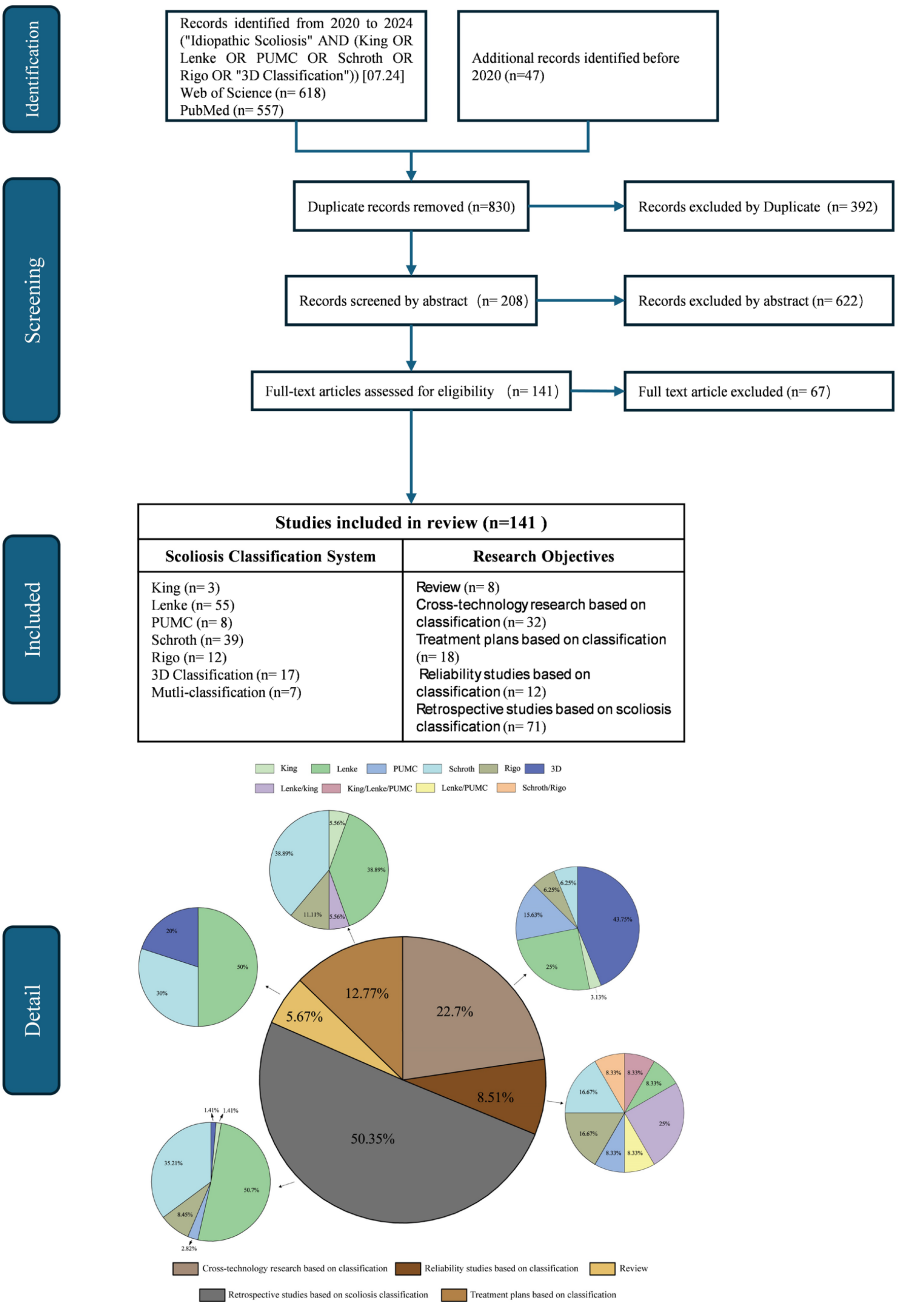
Prisma flow diagram of idiopathic scoliosis classification and research objective.

The literature search was conducted using Web of Science and Science Direct, with search terms such as “King,” “Lenke,” “Rigo,” “Schroth,” “PUMC,” “3D classification,” and “scoliosis,” covering the period from January 2020 to July 2024. For certain classifications, such as King and PUMC, the time frame was extended, while all studies on 3D classification were included. Additionally, relevant literature on the validity of classifications and early foundational studies was supplemented. The content of all selected articles was analyzed, and a total of 141 studies were included in this review, as shown in Figure 1. The number of studies for each classification system is as follows: King classification (3), Lenke classification (55), Schroth classification (39), Rigo classification (12), PUMC classification (8), and 3D classification (17), with7 multi‐classification studies.

In addition to classifying the literature based on the scoliosis classification systems, the studies were further divided into five categories based on research objectives: Review, Cross‐technology research based on classification, Treatment plans based on classification, Reliability studies based on classification, and Retrospective studies based on scoliosis classification. For articles that fall into multiple categories, priority was given based on the order listed above, with preference assigned to earlier research objective. The respective numbers of studies for each category are 8, 32, 18, 12, and 71.

Retrospective studies based on scoliosis classification focus on analyzing the application and performance of different classification systems in historical cases, including statistical analyses of various indications and studies on risk factors associated with different surgical techniques. Treatment plans based on classification investigate how different classification systems are used to develop personalized treatment plans, such as the choice between surgical and conservative treatments. When studies examine the long‐term outcomes of specific surgical techniques, such as the ApiFix Minimal Invasive Dynamic Correction System or the modified Shinshu line for upper instrumented vertebra (UIV) selection, they are prioritized over retrospective studies based on scoliosis classification. Cross‐technology research based on classification explores how emerging technologies, such as computer‐assisted systems and Artificial Intelligence (AI), adopt different classification systems to advance the technological development of scoliosis classification and treatment. All studies on 3D classification are included in this category.

## 3 Surgical Classification

### 3.1 King Classification

**FIGURE 2:**
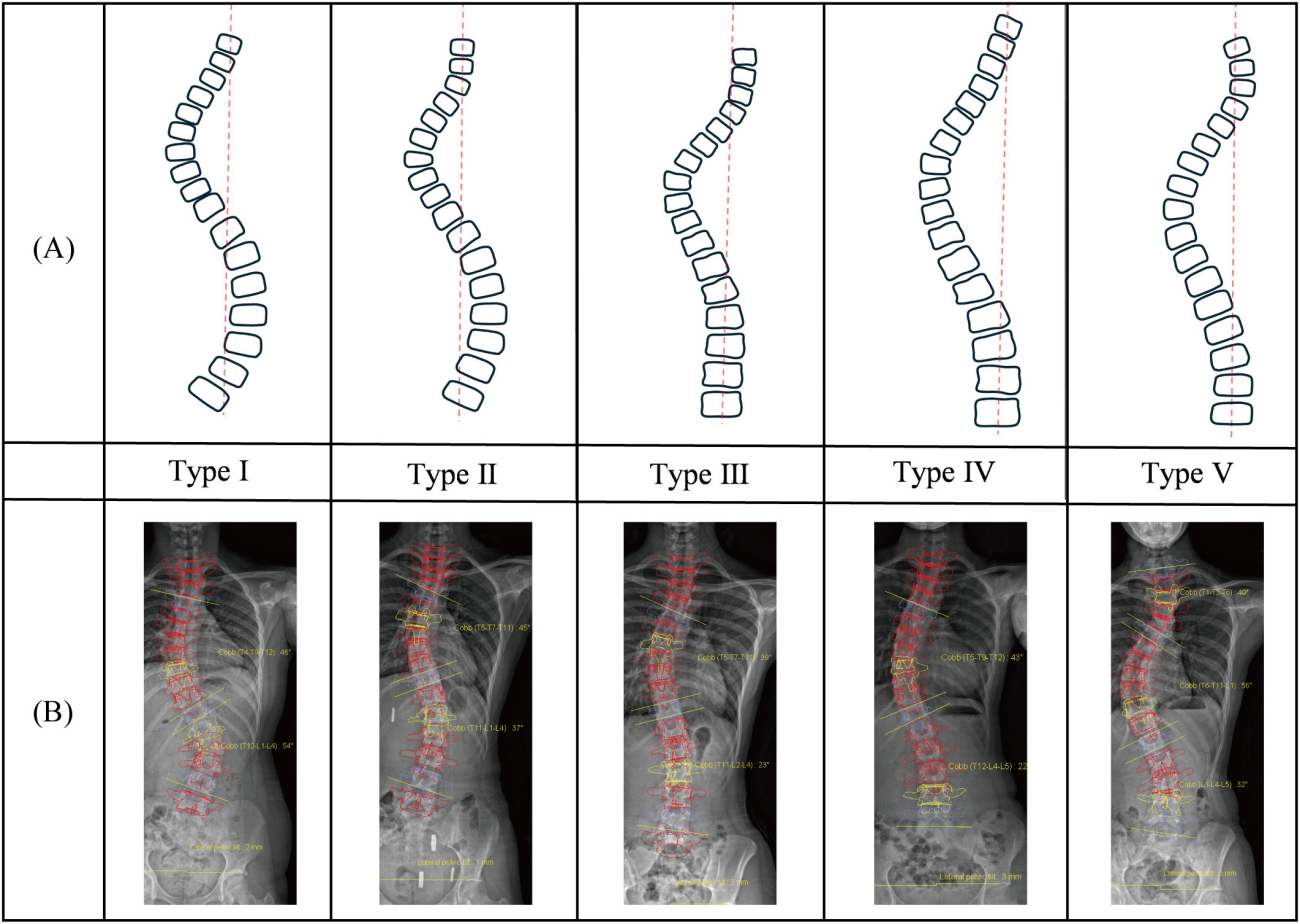
King classification. (A) Schematic diagram of curve types. (B) EOS‐based reconstruction examples.

In 1983, King and Moe established a King classification of IS based on 405 patients who underwent Harrington instrumentation [14]. The classification primarily considers the location of apex vertebra, the severity of the curve, the flexibility, and the presence of compensatory curves. To evaluate the effectiveness of selective fusion, a flexibility index was introduced. This index is calculated by measuring the percentage flexibility of the thoracic and lumbar curves during maximum side‐bending and then subtracting the percentage correction of the thoracic curve from that of the lumbar curve. Additionally, the concept of the stable vertebra was proposed. The stable vertebra refers to the vertebra at the end of the thoracic or lumbar curve that is nearly bisected by the central sacral line, as was shown in Figure 2.

The King classification was revolutionary at the time of its inception. It provided a systematic approach to the treatment of scoliosis. The concepts of the stable vertebra, structural curve, and compensatory curve were also adopted by subsequent classification systems. However, from the perspective of modern medicine, the King classification has significant limitations. One major drawback is that, due to its early introduction, the classification system did not anticipate the shift toward segmental instrumentation systems, which have since become more favored over Harrington rods. As a result, the King classification does not provide reliable guidance for selecting appropriate fusion levels with newer instrumentation techniques.

The interobserver reliability of the King classification system, as indicated by kappa values, ranges from approximately 0.32 to 0.88, while the intraobserver reliability ranges from 0.63 to 0.897 [19, 20, 21, 22]. Overall, the reproducibility of the King classification is considered good, while its reliability is moderate. However, the King classification has notable limitations. It is primarily applicable to thoracic scoliosis and does not account for thoracolumbar double major curves, single thoracolumbar/lumbar curves, or triple curves. Additionally, it does not consider sagittal plane alignment or vertebral rotation in the horizontal plane. The most commonly confused types are King Type III and King Type II.

The high incidence of postoperative decompensation in King Type II classification is primarily attributed to its surgical strategy being limited to coronal correction. In a study by Chen et al., 3 out of 37 patients with King Type II scoliosis developed postoperative trunk imbalance [23]. Similarly, ROYE et al. reported that 9 out of 23 King Type II patients experienced postoperative decompensation, compared to 4 out of 14 King Type III patients [24]. Additionally, in McCall's study, 4 out of 23 King Type II patients developed postoperative decompensation [25].

In recent years, research based on the King classification has been limited, primarily due to its constraints in terms of coverage, consistency, surgical strategy updates, and the widespread adoption of the Lenke classification. Only Trzcińska et al. explored double‐curve IS by classifying it into Type I and Type II according to the King‐Moe classification in two studies [26, 27]. Lee identified patients with left shoulder elevation, Lenke Type 2 or 4, and King Type V as potentially eligible subjects for study [28].

### 3.2 Lenke Classification

**FIGURE 3:**
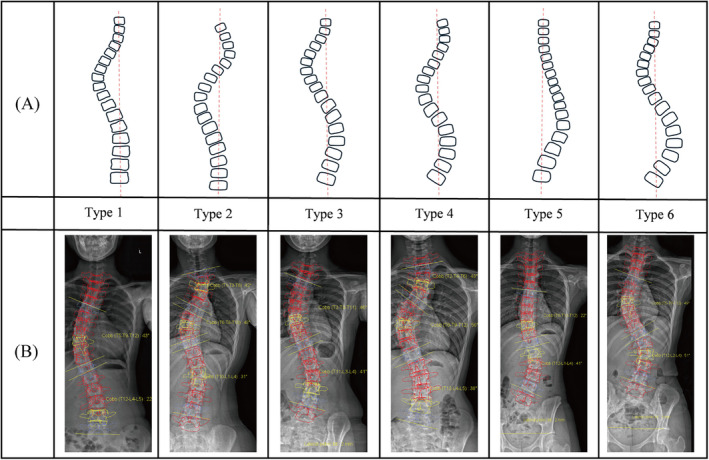
Lenke classification. (A) Schematic diagram of curve types. (B) EOS‐based reconstruction examples.

**FIGURE 4:**
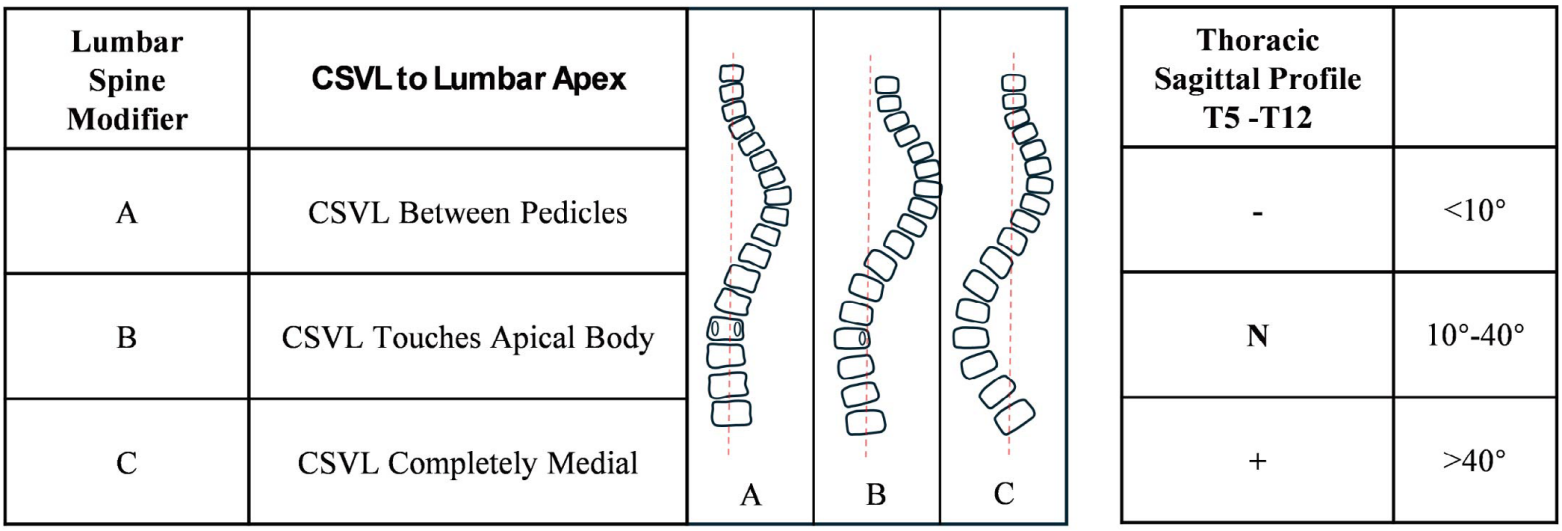
Lumbar spine modifier and thoracic sagittal profile of Lenke classification.

Lenke proposed the classification in 2001, suggesting methods for selecting fusion segments based on different types, as is shown in Table 1 and Figures 3 and 4 [15]. Currently, the Lenke system is internationally recognized as a standard classification.

**TABLE 1:** Curve type of Lenke classification.

Type	Proximal thoracic	Main thoracic	Thoracolumbar/Lumbar	Curve type
1	Non‐structural	Structural (Major)	Non‐structural	Main thoracic (MT)
2	Structural	Structural (Major)	Non‐structural	Double thoracic (DT)
3	Non‐structural	Structural (Major)	Structural	Double major (DM)
4	Structural	Structural (Major)	Structural	Triple major (TM)
5	Non‐structural	Non‐structural	Structural (Major)	Thoracolumbar/Lumbar (TL/L)
6	Non‐structure	Structural	Structural (Major)	Thoracolumbar/Lumbar main thoracic (TL/L‐MT)

The reliability of the Lenke classification system is relatively more complex compared to other systems. The overall interobserver reliability of the Lenke classification is not particularly high, with kappa values ranging from 0.23 to 0.973. This variability is not due to the unreliability of the Lenke system itself, but rather the complexity arising from the curve types, Lumbar Spine Modifier, and Sagittal Thoracic Modifier. Specifically, for curve types alone, the reliability ranges from 0.5 to 0.95, for the Lumbar Spine Modifier it is 0.51 to 1, while the Sagittal Thoracic Modifier has a wider range of variability, with kappa values from 0.41 to 0.734. Intraobserver reliability ranges from 0.46 to 0.796, with curve type reliability between 0.58 and 0.94, Lumbar Spine Modifier reliability from 0.76 to 0.908, and Sagittal Thoracic Modifier reliability from 0.47 to 0.826 [19, 20, 21, 22, 29]. Thomas et al. found that premeasured radiographs can significantly improve reliability, increasing from 0.23 overall, 0.5 for curve types, 0.51 for the lumbar modifier, and 0.45 for the thoracic modifier (non‐measured) to 0.95 overall, 0.95 for curve types, 1 for the lumbar modifier, and 0.93 for the thoracic modifier (measured with training) [22]. Differences in interpretation of the Sagittal Thoracic Modifier and Lumbar Spine Modifier among different observers may contribute to overall significant variability. Therefore, directly comparing the overall reliability with other classification systems is not entirely fair.

Although the Lenke classification provides guidelines for surgical planning, variability still exist in clinical practice. The selection of UIV and lower instrumented vertebra (LIV) is crucial for the success of scoliosis surgery, as it has been proven to effectively prevent coronal decompensation, curve progression, junctional kyphosis, the adding‐on phenomenon, and the need for revision surgery. The literature we reviewed indicates that most studies on the Lenke classification focus on optimizing the selection of fusion segments, yet conclusions remain widely divergent. As demonstrated in Schlager et al.'s study, eight experienced spine surgeons from different hospitals were invited to plan surgeries for 12 representative patients with AIS, revealing significant disagreements in surgical approaches for Lenke types 1, 5, and 6, while there was consistency in the approaches for Lenke types 2, 3, and 4 [30].

Upon organizing the retrieved literature by Lenke subtypes, it was found that Lenke type 1 has the most studies. The main research topics include postoperative alterations of sagittal cervical alignment, shoulder imbalance, adding‐on, lumbar curve correction, and the impact of implant density, anterior spinal fusion and posterior spinal fusion on correction outcomes. Regarding postoperative alterations of sagittal cervical alignment, studies focus on finding evidence of reciprocal sagittal alignment changes after surgery. Mori et al. reported that 73% of patients developed cervical kyphosis within 2 years postoperatively [31]. In a study involving 124 Lenke Type 1 patients, Li et al. identified that 50 patients experienced worsening cervical kyphosis [32]. Several studies have demonstrated that preoperative thoracic kyphosis, cervical kyphosis, and changes in T1 slope are independent factors associated with postoperative cervical hyperkyphosis. Grag et al. found that cervical lordosis is not significantly altered with surgery [33]. Shoulder imbalance is also a major discussion point. Chan et al. classified Lenke Type 1 curve subtypes into flexible and rigid and formulated different UIV selection recommendations to reduce the risk of postoperative shoulder imbalance. In their study, T1 tilt was observed in 41% of patients with rigid Lenke Type 1 curves, compared to 2.0% in those with flexible curves [34]. Akazawa et al. found that patients who underwent surgery with pedicle screws at UIV showed worse shoulder balance, while those with hooks at UIV showed improved postoperative shoulder balance [35]. In terms of implant density, there is currently no established gold standard or definition for low‐density or high‐density constructs. In a study involving 68 patients, Brian et al. reported that ID and pedicle screw density were significant predictors of Cobb angle correction percentage in a multivariate model [36]. Similarly, Mac‐Thiong et al. found that constructs with ID ≥ 90% achieved better correction of the primary coronal curve compared to constructs with ID < 70% [37]. Conversely, many other studies indicate that there is no significant difference in Cobb correction between high‐density and low‐density constructs [38, 39]. Laumonerie et al. further refined the research to focus on sublaminar band density, the number of paired screws divided by the corresponding number of paired anchor vertebrae, suggesting that curve flexibility might be a key factor influencing intraoperative correction [40]. They propose that for flexible Lenke Type 1 AIS, a low sublaminar band density is appropriate. Postoperative distal adding‐on refers to a progressive increase in the number of vertebrae included at the distal end of the primary curve. This condition is characterized by either a deviation of less than 5 mm from the center sacral vertical line to the LIV or more than 5° in the first disc caudal to the LIV [41]. In the study by Li et al., it was found that among 24 patients with a fusion mass shift greater than 20 mm, 17 developed adding‐on, whereas patients with a fusion mass shift less than 20 mm had only a 16.7% probability of developing adding‐on [42]. In Sakai's study, 29% of patients experienced adding‐on, which was significantly associated with postoperative T1 tilt and proximal thoracic‐main thoracic mismatch [43]. Multiple studies have demonstrated that reducing lower instrumented vertebra rotation, T1 tilt, proximal thoracic‐main thoracic mismatch, fusion mass shift, and angle can decrease the incidence of postoperative distal adding‐on. Spontaneous lumbar curve correction refers to the natural alignment improvement of the lumbar curve without direct surgical intervention on the lumbar region. This phenomenon is commonly discussed and studied in the research of Lenke Type 1 and Type 2. Chen et al. reported in their final follow‐up of 51 patients that the thoracolumbar/lumbar curve was spontaneously compensated with a correction rate exceeding 70% [44]. Recent studies have shown that preoperative lumbar spine modifier, lumbar flexibility, sagittal alignment, Risser grade, as well as postoperative thoracic correction, lower instrumented vertebra, and shoulder imbalance are closely associated with spontaneous lumbar curve correction [45]. Studies have shown that rib hump correction and segmental rotation correction are more effective after anterior surgery compared to posterior correction procedures, with coronal correction rates ranging from 51% to 64% [46]. Additionally, anterior surgery has a lower complication rate and demonstrates significant improvements in patients' self‐image, vitality, and mental health, as assessed by the SRS‐22 and SF‐36 questionnaires [47].

Lenke 2 has a major main thoracic curve, with the Proximal thoracic curve being structural. Therefore, Lenke Type 1 and Type 2 are often studied together [31, 44, 45, 48]. The research direction for Lenke 2 is generally consistent with that for Lenke 1 as was discussed above, with a greater emphasis on shoulder imbalance in Lenke 2 compared to Lenke 1. The ratio of proximal thoracic curve and main thoracic curves, upper instrumented vertebra tilt, T2 vertebra rotation ratio, main thoracic Cobb angle, radiographic shoulder height, T1 tilt, Risser sign are closely related to shoulder imbalance [49, 50, 51, 52]. Mimura et al. developed a vertebra selection method, recommending the vertebra first touched proximally by the line between the center of the C7 spinous process and the center of the lowest instrumented vertebra as the upper instrumented vertebra, for the group in which the UIV matched the Modified Shinshu Line Vertebra had a significantly lower prevalence of shoulder imbalance of 23% [53].

Research on Lenke types 3 to 6 is less common than Lenke 1 and 2, with Lenke types 5 being the most frequently studied among types 3 to 6. Lenke type 3 and type 4 curves include the main thoracic curve, with limited flexibility. Lenke type 5 and type 6 curves involve the thoracolumbar/lumbar regions. Challenges in the surgical treatment strategies for Lenke types 3–6 include the selection of the fusion level, managing complications such as Proximal Junctional Kyphosis (PJK) and distal adding‐on, as well as improving long‐term clinical outcomes. Choosing LIV is critical for ensuring postoperative spinal stability and function and must consider both coronal and sagittal alignments [54]. Choosing the LIV is critical for ensuring postoperative spinal stability and function and must consider both coronal and sagittal alignments. Shao et al., placed greater emphasis on the transverse plane, in a study involving 53 patients, they utilized a de‐rotation technique, achieving a final correction rate of 87.1% with a complication rate of 7.6% [55]. For Lenke type 5, the selection of LIV involves identifying the last touching vertebra or stable vertebra to prevent postoperative discomfort and structural imbalances [56]. Distal adding‐on is a common issue in this patient group, which may be related to the selection of LIV and the rotation of the vertebrae [57]. PJK is another significant complication, closely linked to postoperative vertebral stress distribution and intervertebral disc health. Chen et al., in a study of 35 patients, found that the postoperative proximal junctional angle and postoperative thoracic kyphosis are effective predictors of PJK in Lenke Type 5 AIS patients following corrective surgery, with thresholds of 9.45° and 25.25°, respectively [58]. Zhou et al. reported no significant difference in PJK occurrence between high and low pelvic incidence groups. However, in high‐PI patients, PJK was more likely to occur when the UIV was located cranial to the apex of thoracic kyphosis, with an incidence of 31.25%, compared to 4.7% when the UIV was caudal to the apex [59]. Additionally, postoperative biomechanical adjustments may lead to varying degrees of functional recovery, with the angles and extent of correction needing careful management to avoid overcorrection or under correction.

The Lenke classification not only holds extraordinary importance in scoliosis treatment, but also plays a pivotal role in clinical and mechanistic research on scoliosis. In many clinical studies, researchers have utilized the Lenke classification to observe the impact of curve location on muscle, gait, and height. For instance, Luis et al. measured the cross‐sectional area of muscles in different scoliotic types via MRI, finding that, in Lenke types 1 and 2, the cross‐sectional area of the convex side erector spinae and multifidus muscles was significantly larger, while the cross‐sectional area of the quadratus lumborum and psoas major was also markedly larger on the lumbar convex side [60]. Sarah et al. through baropodometric gait analysis, found that gait changes caused by severe scoliosis were somewhat associated with the Lenke classification [61]. Yossi et al. studied factors related to post‐correction height increase in AIS, finding that patients with Lenke types 1 and 2 had significantly lower height gains than those with Lenke types 3, 4, and 6 [62].

In other studies, researchers often focused on specific subtypes to eliminate the overall impact of different classifications. For example, Bai et al. conducted a flexibility assessment on 18 patients with Lenke type 1, finding no significant correlations with age, height, weight, BMI, or Risser sign, but a negative correlation with the initial main thoracic curve Cobb angle [63]. Linda et al. investigated 10‐year pulmonary function outcomes in patients with structural thoracic curves following segmental pedicle screw fixation, finding that preoperative forced vital capacity (FVC) and percentage‐predicted FVC were significantly lower in Lenke type 2 or 4 patients compared to those without structural upper thoracic curves [64]. Mustafa et al. conducted a dedicated study on Lenke type 5, revealing no significant differences in the CSA index of the rectus abdominis, psoas major, multifidus, and erector spinae on the concave and convex sides, but showing that rectus abdominis muscle mass was significantly affected in Lenke type 5 [65]. Sebastien et al. focused on local curve parameters in Lenke types 1 and 5, discovering that curve location influenced coronal and sagittal balance, though abnormal transverse trunk movement remained the same regardless of curve location [66]. Katarzyna et al. examined pulmonary function and scoliosis‐induced body height loss in Lenke types 1 and 3, concluding that in patients with larger Cobb angles, the disparity between predicted and actual pulmonary function parameters was greater, recommending height loss correction in such assessments [67]. Wu et al. found that thoracic deformities in Lenke type 1 affected overall balance control when crossing obstacles, suggesting that signs of increased balance loss risk should be monitored in the management of this patient group [68].

In summary, the Lenke classification is among the primary focal classifications in scoliosis research today. However, as previously described, the Lenke classification was originally designed based on surgical treatment strategies. Although its categorization of structural and non‐structural curves has gained recognition in numerous studies, we believe that in many foundational and biomechanical studies on scoliosis, scientific classifications need to be further refined to facilitate a more comprehensive understanding of scoliosis' effects on the body.

There are many scoliosis classification systems derived from the Lenke classification. Some classification systems primarily aim to address the deficiencies in scoliosis classification across different age groups. Lin et al. proposed a radiographic classification system for adult idiopathic scoliosis based on the Lenke classification [69]. In 2009, Mishiro and Lenke introduced a modified Lenke classification system for IIS and JIS, targeting children from birth to 9 years and 11 months [70]. In IIS and JIS, the main thoracic curve is defined as a minor curve with its apex completely off the plumb line, while the thoracolumbar or lumbar curve is characterized by its apex exceeding the center sacral vertical line. The structural characteristics of the proximal thoracic curve include a Cobb angle greater than 35° and a height difference of over 3 mm in the first ribs when the proximal thoracic Cobb angle is between 10° and 35°. The curve is considered nonstructural when the proximal thoracic Cobb angle measures less than 10°, regardless of the height of the first rib. Sanders et al. modified the Lenke classification to assess maturity and curve progression in girls for conservative treatment, simplifying the process and eliminating the need for additional bending radiographs [71]. Similar to the Lenke classification, the Modified Lenke classification identifies the largest Cobb angle as the primary curve, which is always structural. Secondary curves may be structural or nonstructural, but the classification does not explicitly define the structural characteristics of secondary curves. Thompson et al. defined a secondary curve as structural when its angle is greater than 80% of the primary curve's angle and investigated the impact of this classification on the effectiveness of TLSO bracing [72]. According to their research, they find that thoracic curves have a higher risk of brace treatment failure compared to lumbar curves. The Lenke‐based classification system is currently the mainstream approach for scoliosis classification, even 3D classification systems also being based on Lenke subtypes, which will be elaborated on in the following sections.

With the increasing understanding of surgical correction for AIS, more innovative surgical techniques are being employed for AIS correction, aiming for less invasive procedures and improved therapeutic outcomes. Yi et al. explored the use of minimal invasive lateral lumbar interbody fusion in treating Lenke Type 5 [73]. Metaizeau et al. attempted posterior vertebral body tethering for Lenke 5C curves, though the complication rate appeared to be high [74]. Froehlich et al. investigated the ApiFix Minimal Invasive Dynamic Correction System as an alternative to surgical fusion [75]. However, the long‐term corrective effects require further validation. Several studies on conservative treatments have also utilized the Lenke classification. Widjaja's research found that the Gensingen brace had a positive impact on the curves and chronic low back pain in patients with Lenke 5C curvature patterns [76]. The Lenke classification is also the foundation for the development of new treatment strategies.

The Lenke classification system has extensive applications across interdisciplinary fields. Finite element analysis, as a vital tool for exploring spinal biomechanics, is widely used due to its low cost, ability to precisely simulate complex structures, and high repeatability. Many biomechanical studies of the spine are based on this classification system [77, 78, 79, 80]. AI, which simulates human intelligent behaviors including learning, reasoning, adaptation, perception, and interaction, has demonstrated promising applications in early screening, diagnosis, decision‐making for treatment, intraoperative procedures, and prognosis prediction for scoliosis. Peng et al. developed a prognostic model for postoperative PJK in Lenke 5 patients [81]. Pasha et al. used decision tree to analyze preoperative and early postoperative parameters in selective lumbar fusion surgery to predict the optimal and suboptimal patient outcomes. Sabri et al. developed a scoliosis classification system based on an evolving spiking neural network to assist orthopedic surgeons by photogrammetry images of the human back [82]. Zhang et al. developed an automated Lenke classification system based on X‐ray imaging and the key‐point based detection method [83]. These studies not only enhance our understanding of the applications of the Lenke classification system but also advance the practical application of AI technology in treating spinal disorders, demonstrating its significant potential to improve patient treatment outcomes and increase surgical safety.

### 3.3 PUMC Classification

In 2003, Qiu et al. retrospectively measured the complete radiological data of 427 scoliosis patients. Based on the SRS definitions of scoliosis and the apex of the curve, they incorporated the three‐dimensional deformity characteristics of scoliosis into a classification system and developed the Peking Union Medical College (PUMC) classification system for AIS, which was later recognized by the SRS in 2009 [84].

**FIGURE 5:**
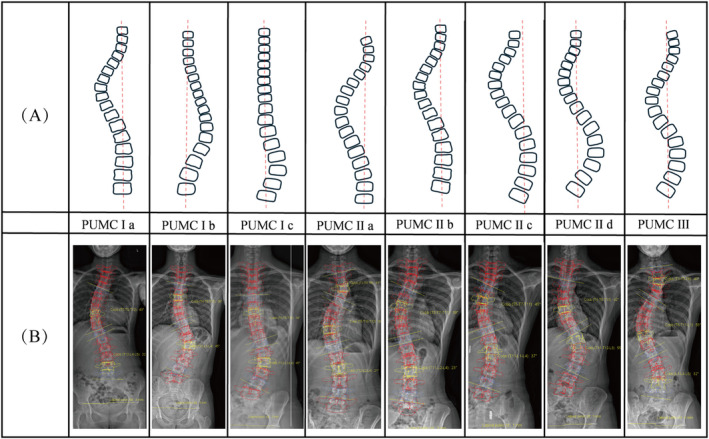
PUMC classification. (A) Schematic diagram of curve types. (B) EOS‐based reconstruction examples.

As shown in Figure 5, the PUMC classification first requires identifying the number of curve apices. A single curve with one apex is classified as Type I, a double curve with two apices as Type II, and a triple curve with three apices as Type III.

Compared to the King and Lenke classifications, the PUMC classification demonstrates stronger reliability, with interobserver kappa values ranging from 0.819 to 0.896, and intraobserver reliability between 0.892 and 0.907 [20, 29]. Zhang et al. developed a computer‐aided system, which increased the average intraobserver and interobserver Kappa values to 0.93 and 0.86 [85]. The higher reliability is attributed to the PUMC classification's basis on the number of curve apices, which aligns well with the clinical characteristics of IS and is easier to remember. Furthermore, the PUMC classification requires fewer angular measurements, reducing the potential for subjective errors. The most frequently confused types are IIb, IIc, and IId. This confusion arises from the close boundary criteria, particularly regarding the Cobb angle and flexibility measurements in the thoracic and thoracolumbar/lumbar (TL/L) curves. However, classification discrepancies in the PUMC system have minimal impact on the surgical fusion range, meaning that even when classification inconsistencies occur, they are unlikely to significantly influence surgical decision‐making.

Currently, the Lenke classification system is widely adopted, while the PUMC classification is less commonly adopted. The advantage of the PUMC system lies in its simplicity, fewer discrepancies, and the fact that nearly half of the cases with discrepancies do not affect the selection of fusion level [86]. In a retrospective analysis of 145 patients, Qiu et al. found that only 2.7% (2/74) of patients whose surgeries fully adhered to the PUMC classification treatment principles developed decompensation, which was lower than the rates observed in patients fully adhering to the King classification (8/68) and Lenke classification (10/80) [87].

However, the PUMC classification system demonstrates certain advantages at the genetic level. Liu et al. identified significant associations of rs225694, rs7774095, and rs2294773 with AIS in northern Chinese patients, with rs225694, rs2294773, rs17861031, and rs6137473 being strongly linked to different PUMC‐classified AIS subtypes [88, 89]. Additionally, in 2019, Zhuang et al. introduced modifications to the PUMC system, resulting in significantly improved interobserver and intraobserver reliability, as well as better postoperative shoulder balance [90]. In the biomechanical studies of spinal fusion treatment for AIS, there are cases where the PUMC classification system has been applied. Wang et al. utilized FEM to investigate the optimal fusion segments for PUMC IId_2 type. The study demonstrated that double‐curve corrective fixation is more effective than single thoracic or lumbar curve fusion [91, 92].

## 4 Non‐Surgical Classification

### 4.1 Lehnert‐Schroth Classification

**FIGURE 6:**
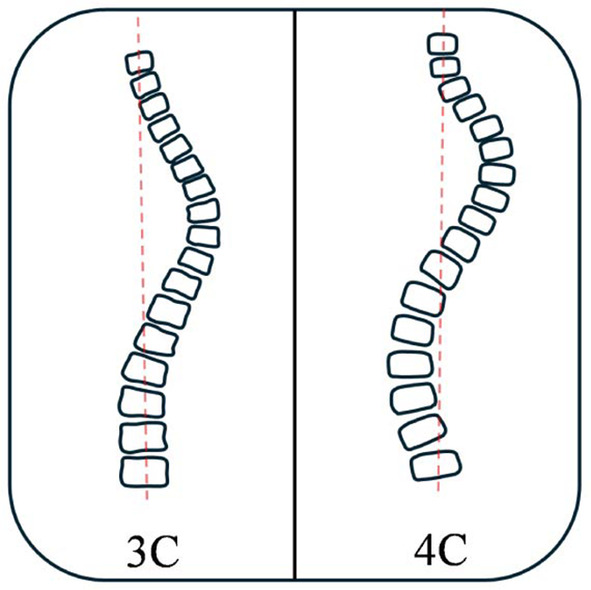
Original Lehnert–Schroth classification.

Lehnert‐Schroth (LS) classification is a widely used classification in the non‐surgical treatment of scoliosis, introduced by Christa Lehnert‐Schroth in the 1970s [17]. In the LS‐classification, the body is divided into different “blocks,” namely shoulder block (SB), thoracic block (TB), lumbopelvic block (LPB) or lumbar block (LB), and pelvic block (PB). As shown in Figure 6, the Original Lehnert‐Schroth classification divides scoliosis into two patterns: three‐curve pattern (3C) and four‐curve pattern (4C). 3C involves the main thoracic curve, with the shoulder‐neck segment, thoracic segment, and lumbo‐pelvic segment presenting twisting and tilting on different planes in radiologic features.4C additionally includes structural curvature in the lumbar or thoracolumbar spine compared to 3C.

**FIGURE 7:**
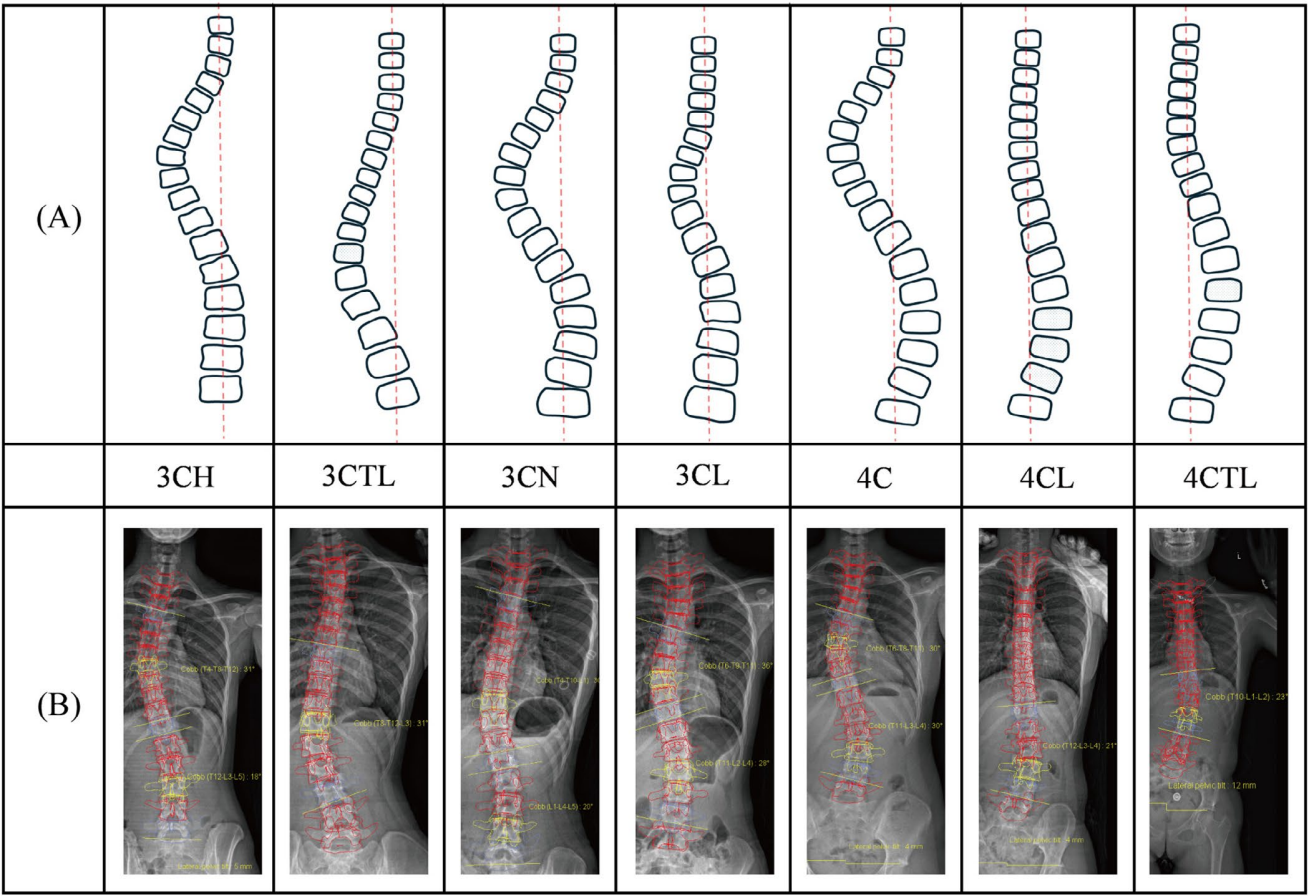
Augmented Lehnert–Schroth (ALS) classification. (A) Schematic diagram of curve types. (B) EOS‐based reconstruction examples.

The Augmented Lehnert‐Schroth (ALS) Classification is a refinement classification based on the Original Lehnert‐Schroth [93]. Although ALS is not sufficient for use for surgery planning, it is adequate when applied to conservative treatment modalities. The individual curvature patterns of ALS are shown in Figure 7.

In treatment of IS, Schroth exercises, a common type of physiotherapeutic scoliosis‐specific exercise, are adjusted according to the classification above [94]. The principles of Schroth exercises include postural awareness, specific muscles training, and breathing techniques, all of which are beneficial for treating scoliosis [95]. Skaggs et al. compared the changes in Cobb angle when standing in a Schroth trained position versus a normal standing position and found that the Cobb angle of the primary curve decreased by an average of 6° in the Schroth trained position [96].

Among the studies involved LS classification, a significant number have now demonstrated the effectiveness of Schroth exercises in treating scoliosis [95, 97, 98, 99, 100, 101, 102, 103, 104, 105, 106, 107, 108, 109, 110, 111, 112, 113, 114]. However, within this series of studies, only two studies specifically mention the detailed ALS classification curvature patterns [97, 106]. Hence, we recommend that future research should put emphasis on the individual curvature patterns and its corresponding treatment exercise for better optimize treatment outcomes.

Besides the retrospective studies above, there are numerous studies that have combined Schroth exercises with other treatment methods, all of which have yielded favorable clinical outcomes like Sling, kinesio taping, spinal manipulation techniques, Electrical muscle stimulation (EMS), Pilates, asymmetric spinal stabilization exercises (ASSE) and brace treatment [115, 116, 117, 118, 119, 120, 121, 122, 123, 124, 125, 126]. Among all the studies, brace treatment is a hot topic of research. The Schroth classification is used to guide the design of Gensingen brace, meaning that different Schroth classifications correspond to different styles of Gensingen brace [126]. Some studies focus on Cheneau and other types of braces with Schroth exercises and achieve favorable outcomes [122, 123, 124, 125].

In addition to the verification and combined treatment research of Schroth, there have been many achievements in biomedical engineering fields which associated to classification. Goral et al. developed a CapsNet and Fuzzy Logic Decision Support System to diagnose scoliosis and plan treatments by Schroth [127]. Similar automated classification systems can significantly reduce doctors' workload and effectively prevent misdiagnosis, this also represents the future direction of automation in healthcare. Weiss et al. conducted a study on bracing using the classification‐based approach for ALS and found the treatment outcomes were superior to those of the bracing based on the finite element modeling approach [128]. Unlike surgical Classification, LS and ALS curvature pattern scan be identified through the appearance of the back. Compared to the radiologic features seen on X‐rays, the appearance of the back is referred to as clinical features. Schreiber developed a Schroth‐curve‐type classification algorithm, according to the algorithm the mean intra‐rater agreement coefficient was 0.64, while experienced raters reached higher estimates with a mean agreement coefficient of 0.81 [129].

In addition to the Original Lehnert‐Schroth and ALS classifications, it is important to note that reliability can be assessed in two ways: one based on the clinical appearance of the back, and the other on X‐rays. This study focuses on the reliability based on X‐rays. The interobserver kappa value for the Original Lehnert‐Schroth classification is 0.71, while the intraobserver value is 1 [130]. The ALS classification is more complex than the Original Lehnert‐Schroth, and Akçay used ICC for reliability analysis, reporting an interobserver value of 0.552 and an intraobserver value of 0.726 [131].

### 4.2 Rigo Classification

The Rigo classification system, developed by Dr. Rigo, was specifically designed for the conservative treatment of scoliosis based on clinical research [132]. The three‐curve and four‐curve classifications, originally proposed by Lehnert‐Schroth and later expanded, were created to define specific correction principles for the use of particular types of braces, such as the Chêneau brace and its derivatives. The goal of this classification is to provide a more precise and reliable diagnosis, facilitating the design of appropriate braces.

**FIGURE 8:**
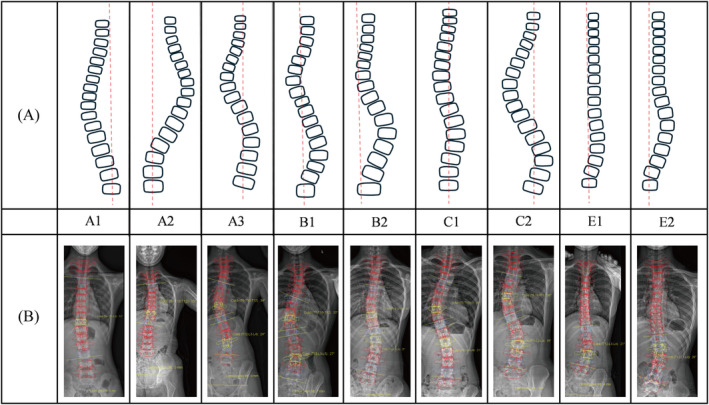
Rigo classification. (A) Schematic diagram of curve types. (B) EOS‐based reconstruction examples.

As shown in Figure 8, the Rigo classification divided scoliosis into four types: Type A (three‐curve scoliosis), Type B (four‐curve scoliosis), Type C (non‐three and non‐four curve scoliosis), and Type E (single lumbar or thoracolumbar scoliosis), with a “D modifier” indicating the presence of an upper thoracic structural curve.

The interobserver reliability of the Rigo classification demonstrates kappa values ranging from 0.7 to 0.942, while intraobserver reliability ranges from 0.87 to 0.912 [132, 133]. Although the Rigo classification is used in the design of Chêneau braces, an interesting study by Zhang et al. compared spine surgeons, radiologists, and orthotists in applying the Rigo classification to scoliosis cases. The results indicated that both interobserver and intraobserver reliability were lower among orthotists compared to physicians [133].

In studies of conservative treatment using corrective exercises, the Schroth classification is commonly adopted, though the Rigo classification is occasionally utilized. For instance, Chongov et al. applied the Rigo classification to assess the impact of corrective exercises on patients [134]. In studies concerning the treatment with Chêneau braces, the Rigo classification is widely employed, as the Chêneau brace is designed based on the Rigo classification for scoliosis [135, 136, 137, 138, 139, 140, 141, 142]. These studies primarily examine the corrective efficacy of the Chêneau brace, particularly emphasizing the importance of 3D in‐brace correction.

## 5 3D Classification

### 5.1 Advantages and Limitations of 2D Classification Systems

In this study, the classification methods that don't require 3D reconstruction are referred to as 2D classifications, as described in the above‐mentioned classification systems. In contrast, classifications that require 3D reconstruction to obtain subtypes are referred to as 3D classifications.

For decades, 2D classifications have accumulated substantial clinical data and provided a valuable resource for advancing the understanding of scoliosis progression and evaluating treatment outcomes, all while not requiring advanced imaging technologies. 2D classifications rely on standard X‐rays, which are both widely accessible and cost‐effective. For instance, the widely adopted Lenke classification plays an important role in guiding clinical decisions and surgical planning.

As the understanding of scoliosis as a three‐dimensional deformity deepens, the limitations of 2D classification systems have become increasingly visible, as AIS is a complex 3D deformity of the spine, involving not only lateral bending but also spinal rotation and torsion. These 2D classifications use parameters from 2D imaging to define a 3D deformity, making it difficult to capture the full 3D nature of scoliosis curves. The SRS has recognized the necessary of developing 3D classification and has authorized the 3D Scoliosis Committee to continue efforts in developing a 3D framework to characterize scoliosis [143].

### 5.2 Prerequisites for 3D Classification Systems

3D classification for scoliosis is not as convenient as 2D classification, and there is still a lack of a clear description of the existing 3D classification systems. Clustering, a statistical tool that groups objects in a dataset into similar clusters, is the main method used in current 3D scoliosis classification [144]. Therefore, 3D classification requires not only 3D reconstruction but also the application of classification algorithms.

In addition to scoliosis‐related 3D reconstructions, techniques like magnetic resonance imaging (MRI) and CT are favored for their high accuracy in modeling geometry. In the field of 3D medical imaging, modeling systems such as Mimics and 3D Slicer are matured, and CT‐based modeling is much simpler compared to X‐ray‐based modeling due to the higher resolution of CT images. These systems significantly reduce the complexity and technical challenges of the modeling process. However, MRI is more commonly used for soft tissue‐related 3D reconstruction. CT has two primary limitations in scoliosis imaging. First, both CT and MRI are typically performed in standing or supine positions, while scoliosis classification is primarily based on standing images. Although CT is used for preoperative 3D reconstruction, there is currently no scoliosis classification based on CT images. It is worth noting that the emergence of standing CT, such as the TSX‐401R (Canon Medical Systems, Otawara, Japan) and the Standing 128 (Qingdao Campo Imaging Medical Technology, Shandong, China), may impact standing 3D reconstructions of the spine, especially for surgical patients. Second, IS predominantly affects adolescents, and excessive radiation exposure should be minimized to protect patients [145]. Therefore, due to the unique characteristics of scoliosis, full‐spine 3D reconstruction based on CT and MRI still has certain limitations.

Biplanar X‐rays and the EOS system are the most used methods for clinical diagnosis and classification of scoliosis. EOS is an innovative low‐dose X‐ray imaging technology originating from France, initially developed for studying scoliosis in children [146]. This technology captures both frontal and lateral X‐ray images simultaneously, and, combined with a 3D reconstruction technique co‐developed by the Biomechanics Laboratory of the National Conservatory of Arts and Crafts (ENSAM, Paris) and the Imaging and Orthopedic Research Laboratory (LIO, Montreal), provides detailed information on the patient's spine and skeletal structure [147]. Since its commercialization in 2007, EOS technology has been widely adopted in clinical practice. Its ability to deliver lower radiation doses while offering high‐quality images allows for more accurate skeletal and spinal assessments, which are crucial for diagnosing and treating orthopedic and spinal conditions [148]. For traditional biplanar X‐rays, due to the lack of open‐source software, 3D reconstruction remains a challenge for most researchers unless they invest in acquiring or developing commercial software and equipment. Known reconstruction software includes Spine 3D and Clindexia, which are based on feature points and shape fitting principles.

Other emerging technologies, such as surface scanning, ultrasound, and Forethought, hold great potential for clinical application. However, since most clinicians still rely on traditional radiographic imaging for scoliosis classification, these new technologies have yet to gain widespread use in scoliosis type classification.

### 5.3 Current 3D Classification System

After screening the literature, a total of 13 studies on 3D classification systems for patients with AIS were identified, as shown in Tables 2 and 3. All classification systems are based on biplanar X‐rays and the EOS system. K‐means clustering and its derivative algorithms are the most commonly used classification methods.

**TABLE 2:** Characteristics of included 3D classifications systems.

Author	Algorithm	Equipment	Number of patients	Curve type	Number of subgroups	Number of parameters
Arginteanu et al. [149]	K‐means clustering	EOS	30	Lenke 1	2	2
Sangole et al. [150]	ISOData unsupervised clustering	X‐ray	172	Lenke 1	3	4
Duong et al. [151]	Fuzzy k‐means clustering	X‐ray	409	Not specified	5/12	20
Bernard et al. [152]	No	EOS	63	Lenke 1A	2	3
Pasha et al. [153]	No	X‐ray	40	Lenke 1	5	Not mentioned
Kadoury et al. [154]	Non‐linear manifold embedding algorithm and k‐means clustering algorithm	X‐ray	170	Lenke 1	4	Not mentioned
Shen et al. [155]	Fuzzy c‐means clustering algorithm	X‐ray	952	Not specified	11	10
Garcia‐Cano et al. [156]	Dynamic ensemble selection	X‐ray	962	Not specified	3	8 (features)
Thong et al. [157]	K‐means clustering	EOS and X‐ray	663	Not specified	11	714
Pasha et al. [158]	K‐means clustering	X‐ray	67	Right thoracic types	3	1
Pasha et al. [159]	Fuzzy C‐means clustering	EOS	371	Not specified	9	102
Pasha et al. [160]	Agglomerative hierarchical clustering	EOS	103	Not mentioned	5	15
Pasha et al. [161]	No	EOS	141	Lenke 1	2	4

**TABLE 3:** Characteristics of included 3D classifications systems.

Author	Cobb	PMC	ARV	TK	LL	EX
Arginteanu et al. [149]						Spinal centerline twist Spinal centerline writhe
Sangole et al. [150]	√	√	√	√		
Duong et al. [151]						Wavelet approximation coefficients describing 3D spinal curves
Bernard et al. [152]	√					Pelvic incidence Pelvic tilt
Pasha et al. [153]						
Kadoury et al. [154]						Simplified 3d‐dimensional representation of 3D spine models
Shen et al. [155]	√	√	√	√	√	
Garcia‐Cano et al. [156]			√			Cartesian coordinates First and second derivatives of the centroid Leave‐n‐out angles Fan leave‐one‐out angles
Thong et al. [157]						All the anatomical landmarks
Pasha et al. [158]						Coordinates of the vertebrae
Pasha et al. [159]	√	√	√	√	√	3D alignments, frontal tilt, sagittal tilt, axial rotation, vertebral centroids et al. 102 variables per spine
Pasha et al. [160]	√	√	√	√	√	Pelvic incidence Sacral slope Pelvic tilt Pelvic obliquity
Pasha et al. [161]						Writhe Torsion

Abbreviations: ARV, apical rotation value; LL, lumbar lordosis; PMC, plane of maximum curvature; TK, thoracic kyphosis.

Research on the 3D classification of scoliosis is mainly divided into two directions. The first is the overall classification of scoliosis. For example, Duong et al. conducted a fuzzy k‐means classification study on 409 scoliosis patients, dividing scoliosis into 5 or 12 categories based on 20 clinical indicators [151]. The 5‐category system is similar to the Lenke and King classifications, while the 12‐category system is more comprehensive, subdividing the 5‐category system into subgroups according to variations in other planes of spinal deformity. As shown in the table, other studies have classified scoliosis into 11 subtypes and 9 subtypes, respectively [155, 157, 159]. The emergence of these different classifications is complex and does not necessarily imply that more categories result in a more refined system. In addition to the inherent differences in scoliosis among patients, a significant part of the variation arises from the use of different parameters. Pasha et al. using 102 parameters, classified scoliosis in 371 patients into 9 subtypes [159]. Thong et al. in a study of 663 patients, initially formed a vector of 714 based on the anatomical points of the vertebral body, dividing scoliosis into 11 subtypes [157].

Another type of research focuses on further analyzing subtypes based on specific scoliosis classifications. Currently, a research hotspot is the analysis of Lenke Type 1 scoliosis, with the primary goal of optimizing surgical planning, such as internal fixation segments, correction methods, and fusion segment selection. This study also supports the development of 3D classification systems. Sangole classified 172 Lenke Type 1 curves using ISOData clustering analysis, resulting in three subtypes [150]. Kadoury et al. classified Lenke Type 1 scoliosis into four subtypes using a non‐linear manifold embedding algorithm and k‐means clustering. Some categories of research utilize mathematical principles to describe real 3D parameters for classification [154]. For instance, Arginteanu et al. applied the Călugăreanu–White–Fuller principle and classified Lenke Type 1 scoliosis into two subtypes based on spinal centerline twist and spinal centerline writhe [149]. Pasha further supplemented the current AIS classification by dividing scoliosis into two subtypes based on writhe and torsion, conducting comparative studies [161]. This classification method not only effectively describes scoliosis subtypes but also quantifies the deformity, making generative scoliosis modeling possible. It holds great potential for future scoliosis research, particularly in brace treatment. In addition, Saba et al. attempted to use biplanar X‐rays to identify these 3D subtypes, and their results indicated that radiologists and orthopedic surgeons were able to recognize the 3D subtypes of Lenke Type 1 AIS from patients' X‐rays with moderate to high reliability [153]. This suggests that, in the future, certain 3D classifications may be applicable without quantitative 3D image post‐processing.

Currently, there are relatively few clinical studies based on 3D classification. Pasha conducted a retrospective analysis of 76 right thoracic AIS patients who were followed up for 2 years and found that the selection of the UIV and LIV had different impacts on surgical outcomes across the five subtypes [162]. In addition to revealing significant differences in fusion length and the rates of radiographic adverse outcomes, the study found that 3D classification Type 3, characterized by hypokyphosis, vertebral rotation changes in the thoracolumbar region with an S‐shaped axial view, and Type 4, characterized by a flat or lordotic sagittal profile, positive SVA with a V‐shaped axial view, had the highest rates of suboptimal frontal balance and PJK development.

To date, 3D classification systems for AIS patients have been receiving increasing attention due to their significance in assessing the severity and progression of deformities. These systems aim to determine optimal strategies for both surgical and conservative treatments. However, the following challenges remain:

1. More complex classification systems may require advanced equipment, and some clinicians may not yet be prepared to adopt new, complex, and costly technologies that could alter their clinical practices.
2. While establishing a 3D surgical classification for AIS by identifying all essential curve information observed in 2D imaging has been initially deemed feasible, its reliability remains low.
3. Many 3D classification systems are based on Lenke subtypes; however, there is currently no consensus on which classification method should be adopted for conservative treatment. Direct application of 3D classification systems may overlook spinal flexibility, but this aspect can potentially be incorporated into 3D classifications through additional measures.
4. 3D classification systems are still in their early stages, with limited research linking them to clinical treatment outcomes. Their impact on improving therapeutic efficacy remains unclear and warrants further investigation.
5. The extraction of appropriate key features during 3D reconstruction is yet to be standardized. Variations in training datasets, algorithms, and parameters can lead to inconsistent classification results, ultimately reducing reproducibility.

## 6 Discussion

### 6.1 Characteristics and Evolution of 2D Classification

An ideal classification system for AIS should encompass all curve patterns without being overly complex, be easy to remember, and demonstrate strong intra‐ and inter‐observer reproducibility. It should be treatment‐focused and outcome‐driven, providing appropriate treatment strategies for each type, and offer scope for future expansion.

The King classification primarily targets thoracic curves, has limited coverage, and is not suitable for modern complex cases. Although the classification structure is relatively simple and easy to remember, it has poor inter‐observer agreement, particularly with complex curves, and limited reproducibility. It is best suited to older surgical techniques and has little relevance in current surgical decision‐making. Additionally, it lacks scope for further development and has been phased out.

The Lenke classification covers various spinal curve types and distinguishes structural from non‐structural curves, making it suitable for surgical planning and widely used in clinical practice. It has high overall reliability, but discrepancies may arise among observers, particularly regarding the lumbar spine modifier and sagittal profile modifier. As surgical outcomes accumulate across numerous cases, more refined subtypes and corresponding treatment plans have led to improved therapeutic outcomes. Therefore, the Lenke system has further expansion potential, effectively guiding surgical fusion segment selection and supporting surgical decision‐making, with its application gradually extending to patients undergoing conservative treatment.

The PUMC classification covers a range of curve types and is particularly useful for assessing deformities in different spinal regions. It is intuitive and well‐structured, although the numerous subtypes may be more challenging to memorize. Studies indicate that the PUMC classification has good reliability, with consistency notably enhanced by computer‐assisted technology. This classification supports surgical planning and is especially applicable to patients undergoing surgical treatment, as it facilitates individualized decision‐making. Its advantage lies in the fact that minor classification discrepancies do not substantially impact surgical decisions or outcomes. However, its structure‐based classification limits its potential for future development.

In surgical treatment, the King, Lenke, and PUMC classification systems each define fusion ranges for different scoliosis types. The King classification is limited in guiding current three‐dimensional corrective approaches, particularly in King Type II cases. Many studies have further refined new subtypes and treatment strategies based on the Lenke classification, paving the way for its increasing effectiveness in guiding scoliosis surgery. For example, Lenke initially recommended setting the UIV for the main thoracic curve at T3, T4, or T5. However, with further research, Chan and Cho further subdivided Lenke Type 1 based on flexibility and L4 tilt, respectively [9, 34]. Some studies also employ 3D classification systems to further optimize Lenke‐based treatment plans. The PUMC classification emphasizes preserving more motion segments to reduce surgical trauma and provides clear guidance on fusion levels. Although less widely used, studies based on the PUMC classification have reported lower incidence rates of trunk imbalance.

The Schroth classification addresses different functional curve patterns and is widely applied in conservative treatments, particularly for designing posture correction strategies. It has a certain level of reliability in the conservative treatment domain but is heavily dependent on the expertise of physical therapists. The system is based on functional descriptions, making it relatively easy to memorize, with good intra‐observer reproducibility, though inter‐observer consistency may be affected by differences in therapist experience. The Schroth classification has room for development, especially in the context of personalized rehabilitation plans.

The Rigo classification encompasses various curve patterns and is focused on conservative treatment, especially with respect to brace application, showing a high level of practicality. Its structure is relatively complex but provides valuable guidance for experienced brace designers and physical therapists. The Rigo classification demonstrates high intra‐observer agreement, though inter‐observer consistency may vary due to differences in professional experience. Primarily aimed at brace correction, it provides strong guidance for non‐surgical treatment plans and shows significant potential for expansion, particularly when integrated with 3D technology and computer‐assisted correction methods.

In conservative treatment, the Rigo classification is widely used in brace design, with the Chêneau brace being a representative model. Similarly, the Schroth classification is mainly used in physiotherapy. Both braces are types of thoracolumbosacral orthoses (TLSO), with the Gensingen brace being derived from the Chêneau brace. The Chêneau brace emphasizes principles of three‐dimensional correction, including three‐point support, regional rotation, sagittal alignment, and balance. In contrast, the Gensingen brace focuses on rapid production with reduced risk of pressure sores and simplifies classification in its design, primarily for use in physiotherapy. However, since conservative treatment for scoliosis is not limited to bracing, there is currently no research systematically comparing the therapeutic efficacy of braces based on these two classification systems.

With the advancement of computer‐assisted technologies, current image reading has gradually transitioned from manual recognition to AI‐based recognition. The development of computer vision and AI has significantly improved the accuracy of scoliosis classification, reducing errors caused by human factors. With the rapid development of computer‐assisted technologies, computer vision, artificial intelligence (AI), and big data analysis are gradually being integrated into the diagnosis and classification of scoliosis. This trend holds great potential, not only significantly improving the accuracy and consistency of classification but also reducing errors caused by human factors. For example, Zhang et al. developed a computer‐assisted system that increased the intraobserver and interobserver Kappa values of the PUMC classification to 0.93 and 0.86, respectively [85]. Lu et al. developed an intelligent AI system, which demonstrated high accuracy and excellent reliability compared to senior physicians showed excellent reliability compared to senior physicians (ICC 0.962) [163]. Thus, the advancement of computer‐assisted technologies can effectively improve classification accuracy and reduce physicians' workload.

### 6.2 Research Progress Based on 2D Classification

In practical research, the Lenke classification is the most frequently used, while the Schroth method is more commonly applied in conservative treatments. The development of 3D classifications is still in its early stages, and the King classification is gradually fading from the research landscape, with only two related studies in the past 5 years. Research based on the Lenke classification, in addition to retrospective studies and the exploration of new surgical methods, has also examined its correlations with muscle structure, gait, height changes, and lung function. Although the classification of structural and non‐structural curves in Lenke has been widely recognized, it is important to note that the Lenke classification was originally designed based on surgical treatment strategies, and we believe that the classification itself may not be directly related to the above research themes. Furthermore, in FEA studies on scoliosis, although the Lenke classification is noted for patients, material parameters specific to structural and non‐structural curves have not been further refined in these studies. The PUMC classification system has demonstrated research potential in the fields of genetics and FEA, though the number of studies based on PUMC remains relatively low. Research on the Schroth method focuses primarily on combining Schroth exercises with other treatment approaches to enhance therapeutic outcomes, while the Rigo classification is mainly used in studies on brace design. Currently, AI shows promising applications in early screening, diagnosis, treatment decision‐making, intraoperative procedures, and prognosis prediction for scoliosis. Among the studies we reviewed, both the Lenke classification and the Schroth method involve AI applications, which may become research focal points in the future.

### 6.3 The Necessity and Progress of 3D Classification

From a geometrical perspective, in scoliosis accompanied by kyphosis, the rotation of the apex vertebra is, in some sense, opposite to the rotation of the plane of maximum curvature. Furthermore, when the degree of kyphosis is relatively small, the rotation of the plane of maximum curvature is at its greatest. There are similarities and differences in the recognition of scoliosis using 2D classification systems. For instance, it is not uncommon for two distinctly different curves to have the same Cobb angle. Similarly, curves with similar coronal and sagittal profiles may appear entirely different in an axial view. Classifications based on one or two anatomical planes fail to capture the true pattern or severity of spinal deformities. A three‐dimensional perspective provides valuable data, including axial views of the spine, intervertebral rotation at each segment, vertebral wedging differences, and the torsion at the point of maximum curvature. Research by Nault et al. suggests that the three‐dimensional morphology of the spine may predict deformity progression [164]. In surgical treatment, scoliosis management has evolved from an initial focus on coronal correction to Lenke's emphasis on three‐dimensional strategies, and now, further attention is being paid to de‐rotation. Similarly, in conservative treatments, the Milwaukee brace initially focused on spinal elongation, whereas subsequent designs, such as the earlier Boston brace, emphasized two‐dimensional corrections. Modern braces, like the Chêneau and Gensingen braces, now prioritize three‐dimensional corrections. The historical development of AIS treatment has consistently progressed from 2D to 3D approaches. Similarly, the evolution of 3D classification systems has followed a comparable trajectory, moving from virtual 3D classification based on 2D data, to 3D classifications based on reconstructed images, and now to classifications incorporating true geometric torsion of the entire spine.

Thus, 3D classification systems hold significant value in assessing the severity and progression of scoliosis. Further research into 3D classification could facilitate the standardization of brace and surgical treatments.

## 7 Conclusion

This study reviews different scoliosis classification systems, tracing the development from traditional 2D classifications, such as the King, Lenke, and PUMC classifications, to emerging 3D classification systems. Although 2D classifications still play an important role in surgical planning, their limitations are increasingly evident. With rapid advances in imaging technology and AI, 3D classification systems are being used more widely, providing a more complete assessment of spinal deformities and improving the accuracy of both surgical and conservative treatment planning. However, integrating 3D classification systems into routine clinical practice remains challenging, requiring specialized equipment, software, and handling of complex models. Future research should focus on optimizing 3D classification systems to make them more practical for clinical use while ensuring simplicity and ease of application. Additionally, as technology advances, combining enhanced 2D methods with 3D approaches or adapting 3D classifications for 2D applications may represent an ideal way forward, providing stronger support for personalized scoliosis treatment, ultimately improving patient outcomes.

## Author Contributions

All authors meet the authorship criteria in accordance with the latest guidelines of the International Committee of Medical Journal Editors (ICMJE), and all authors have approved the final version of the manuscript. All authors had full access to the data in the study and take responsibility for the integrity of the data and the accuracy of the data analysis. **Wenqing Wei:** conceptualization and writing – original draft. **Yaolong Deng:** writing – review and editing. **Fang Xie:** writing – review and editing. **Junlin Yang:** supervision. **Liang Cheng, Yating Dong, Jiale Gong, Tianyuan Zhang:** investigation. All authors have read and agreed to the published version of the manuscript.

## Conflicts of Interest

The authors declare no conflicts of interest.
